# Predation Success By A Plant-Ant Indirectly Favours The Growth And Fitness Of Its Host Myrmecophyte

**DOI:** 10.1371/journal.pone.0059405

**Published:** 2013-03-14

**Authors:** Alain Dejean, Jérôme Orivel, Vivien Rossi, Olivier Roux, Jérémie Lauth, Pierre-Jean G. Malé, Régis Céréghino, Céline Leroy

**Affiliations:** 1 Université de Toulouse, UPS, Ecolab, Toulouse, France; 2 CNRS, Écologie des Forêts de Guyane (UMR-CNRS 8172), Kourou, France; 3 CIRAD, Ecofog (UMR-CIRAD 93), Kourou, France; 4 IRD, Maladies Infectieuses et Vecteurs, Écologie, Génétique, Evolution et Contrôle (UMR- IRD 224), Bobo-Dioulasso, Burkina Faso; 5 CNRS, EDB (UMR-CNRS 5174), Toulouse, France; 6 CNRS, Laboratoire d'Ecologie Fonctionnelle (UMR-CNRS 5245), Toulouse, France; 7 IRD, AMAP (botAnique et bioinforMatique de l'Architecture des Plantes; UMR-IRD 123), Montpellier, France; Royal Holloway University of London, United Kingdom

## Abstract

Mutualisms, or interactions between species that lead to net fitness benefits for each species involved, are stable and ubiquitous in nature mostly due to “byproduct benefits” stemming from the intrinsic traits of one partner that generate an indirect and positive outcome for the other. Here we verify if myrmecotrophy (where plants obtain nutrients from the refuse of their associated ants) can explain the stability of the tripartite association between the myrmecophyte *Hirtella physophora*, the ant *Allomerus decemarticulatus* and an Ascomycota fungus. The plant shelters and provides the ants with extrafloral nectar. The ants protect the plant from herbivores and integrate the fungus into the construction of a trap that they use to capture prey; they also provide the fungus and their host plant with nutrients. During a 9-month field study, we over-provisioned experimental ant colonies with insects, enhancing colony fitness (i.e., more winged females were produced). The rate of partial castration of the host plant, previously demonstrated, was not influenced by the experiment. Experimental plants showed higher δ^15^N values (confirming myrmecotrophy), plus enhanced vegetative growth (e.g., more leaves produced increased the possibility of lodging ants in leaf pouches) and fitness (i.e., more fruits produced and more flowers that matured into fruit). This study highlights the importance of myrmecotrophy on host plant fitness and the stability of ant-myrmecophyte mutualisms.

## Introduction

Mutualisms, defined as cooperative interactions between species where each partner derives a fitness benefit, are based on “invested benefits” corresponding to an adaptation by each species to obtain benefits from its partner with the return exceeding the costs of the investment [1]–[4]. Such context-dependent outcomes vary according to the interacting partners over space and time, thus influencing the evolutionary fate of a mutualistic relationship and providing information on how and when mutualisms arise, persist, and vanish [5].

When hosts transmit symbionts by “vertical transmission” to their offspring the mutualisms are evolutionarily stable; yet, most mutualistic interactions are transmitted “horizontally” as the partners disperse separately. In the latter case, the reproduction of each partner might be subject to a trade-off if the resources invested by one partner in its own reproduction are lost for the other partner. Such a trade-off is a major source of instability as the symbionts may evolve traits promoting a reduction in cost, engendering the emergence of “cheaters” obtaining benefits at minimal cost [6]. Over time, they end up by completely sterilizing their hosts [7],[8].

The subsequent instability would turn every mutualist into a parasite if not counterbalanced by specific conditions such as partner selection and fidelity, spatial structure (i.e., limited dispersal), and, above all, retaliation against exploiters [1],[7],[9]–[13]. Because mutualisms are stable and ubiquitous in nature, the intrinsic traits of one partner can generate an indirect and positive outcome for the other, resulting in “byproduct benefits”. The resulting absence of cost (or very low cost) permits an equilibrium between the costs and benefits to be easily established between the partners [14]–[16].

Ant relationships with myrmecophytes, or plants housing a limited number of so-called ‘plant-ants’ in domatia (i.e., hollow branches or thorns and leaf pouches), are interesting models for studying conflicts and breakdown within mutualisms. Also, myrmecophytes usually provide their guest ants with food, particularly extrafloral nectar (EFN) and/or food bodies (FBs), while, in return, plant-ants protect them from herbivores, competitors and pathogens [17] through their intrinsic predatory, territorial and cleaning behaviours. So, these protections correspond to byproduct benefits for the host myrmecophytes (see [18]). The same is true when ants provide their host plants with nutrients (e.g. prey remains and faeces) that accumulate in the domatia. These nutrients are absorbed through the rhizomes, roots, protuberances, or the walls of the domatia. This phenomenon, called myrmecotrophy, has been noted for epiphytes and for some phanerophytes adapted to the nutrient-poor, lateritic soils of tropical rainforests [17],[19].

The size of plant-ant colonies can be limited by the availability of space and food. As the host myrmecophyte grows, the guest colonies have more nesting space thanks to a greater number of domatia and so can grow in turn, while the production of EFNs and FBs increases. Yet, certain plant-ant species are somewhat less dependent on their host myrmecophyte to provision them because they tend hemipterans to obtain honeydew and/or are predators [17]. Mutualisms between myrmecophytes and plant-ants are transmitted horizontally, so that the energy invested by the myrmecophytes in producing flowers and fruits is not allocated to producing greater nesting space for the ant colonies. Reciprocally, winged ant sexuals are not involved in protecting the host plant foliage. This gives rise to a conflict of interest between the partners. By destroying flowers, the ants “sterilize” the plant and trigger the reallocation of host-plant resources from reproduction to vegetative growth. The sterilization is partial; otherwise, the ant species are considered parasites of the mutualism [13],[17],[20]–[24].

**Figure 1:**
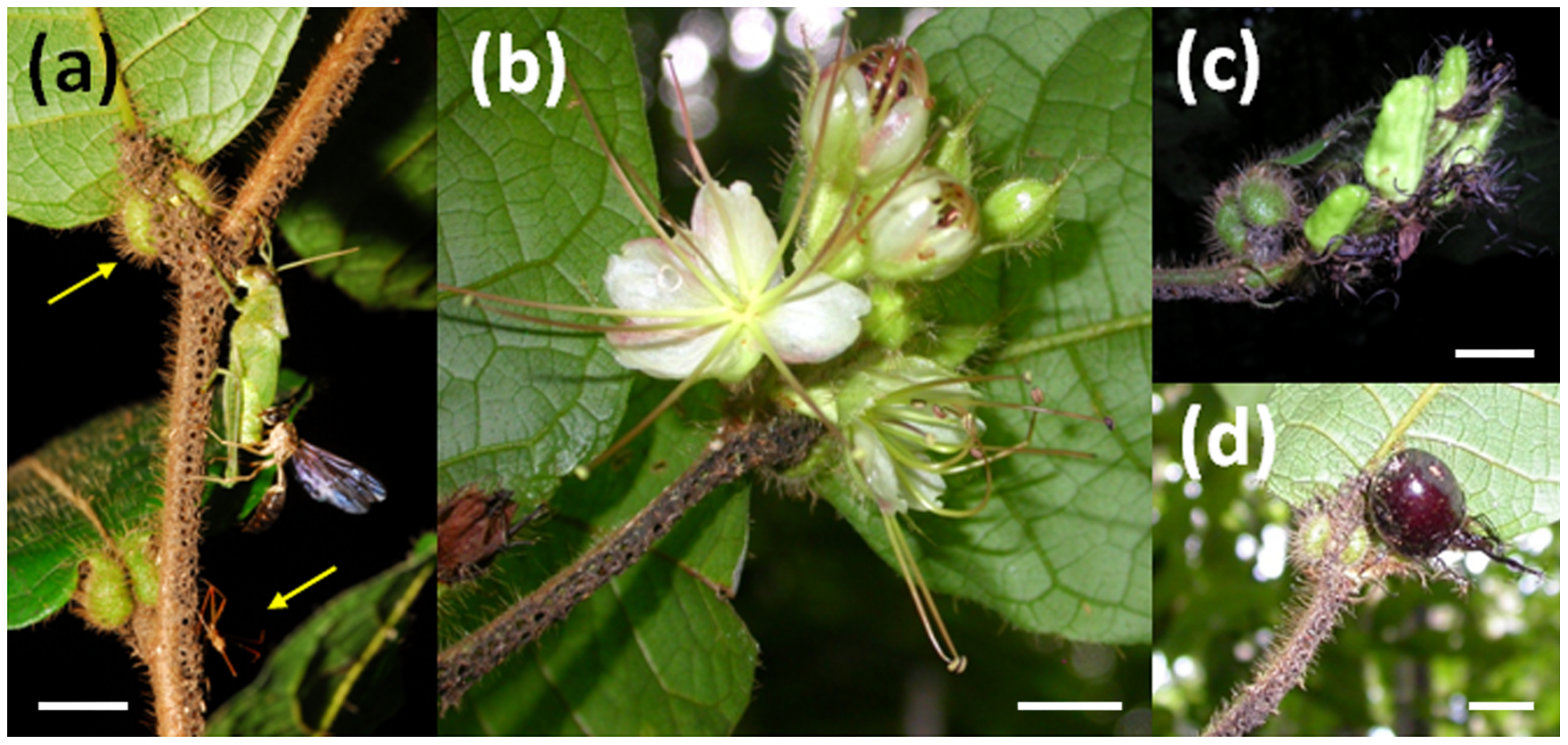
*Hirtella physophora* leaves, flowers and fruits. (a) Leaves bear leaf pouches (left arrow) at the base of their laminas. *Allomerus decemarticulatus* workers capture a green locust thanks to their trap: a gallery made using severed host plant trichomes and the mycelium of an Astomycota fungus that the ants manipulate to create a composite material pierced by numerous holes (from under which the workers ambush prey). A wasp is seen robbing a piece of the locust abdomen; the wasp was also captured in turn as was the red Reduviid (right arrow). (b) At the distal position of the branch, flowers are segregated on racemous inflorescences at different stages of maturation from flower buds to fully open flowers. (c) Development of young, green (i.e. unripe) drupes. (d) A dark purple (i.e. ripe) drupe. Scale bars represent 1 cm.

We focused this study on *Hirtella physophora* (Chrysobalanaceae) that houses colonies of *Allomerus decemarticulatus* (Myrmicinae) in pouches situated at the base of the leaf lamina (Fig. 1) and provides them with extrafloral nectar. Workers build galleries under the stems of their host-plants that serve as traps to capture insects of up to 1800 times the weight of a worker (Fig. 1; [25]). To build these galleries, the workers first cut plant trichomes along the stems, clearing a path; then, using uncut trichomes as pillars, they build the vault of the galleries by binding together the cut trichomes with the mycelium of a fungus that they manipulate [25]. This third partner, an Ascomycota from the order Chaetothyriales, therefore serves a structural purpose. *Allomerus decemarticulatus* also supply their host tree with nutrients via the walls of the domatia and the fungus with wastes, whilst the fungus, in turn, also provides the host plant with nutrients [19],[26]. Finally, the workers partially castrate their host plant by cutting and chewing both the sterile and fertile parts of the flower buds [24],[27].

We hypothesized that when particularly successful in catching prey, mutualistic ant colonies increase their own fitness by producing more sexuals while also providing benefits to their host plant. Indeed, the more prey they capture, the more they provide the host plant with nutrients that then has greater growth (more leaves equals more housing available for guest ants) and fitness (more flowers and fruits produced in spite of the partial castration).

## Materials And Methods

### Ethics Statement

This study was conducted according to relevant national and international guidelines. Sample collections necessary to scientific research were authorized by the French *Office National des Forêts* (*ONF*) provided that their impact upon the environment is considered negligible (details of the permit in [28]).

### Study Site And Model

This study was conducted between November 2007 and July 2008 on the top of a hill (05° 03.697′ N; 52° 58.620′ W – 05° 03.638′ N; 52° 58.612′ W) situated in the pristine forest near the field research station at Petit Saut, Sinnamary, French Guiana. *Hirtella physophora*, an understory plant found in pristine Amazonian forests and strictly associated with *A. decemarticulatus* in the study area, has long-lived leaves that bear a pair of leaf domatia at the base of each lamina; the flowers are segregated on racemous inflorescences and produce dark-purple drupes (Fig. 1). These trees have a much longer lifespan (up to ca. 350 years) than their associated ant colonies (ca. 20 years) [27].

### Over-Provisioning The Ants

We investigated the role of the ants in the nitrogen provisioning of their host plant by providing the colonies of experimental *H. physophora* with surplus prey twice a week for 9 months (colonies normally capture one to two prey items per week [25]). Captured at a light trap situated in Petit Saut, the prey – large moths and grasshoppers – were cut into pieces and the thoraxes plus legs (ca. 1g) were then provided to the ants by holding them close to the galleries where the workers immediately seized and dismantled them [25]. The 41 control and 31 experimental *H. physophora* trees selected for the main experiment were of similar height, trunk diameter at their base and number of leaves at the beginning of the experiment; height: 1.4±0.09 m *vs*. 1.35±0.08 m (*t* = 0.80; *df*  = 70; *p* = 0.42), trunk diameter at the base of the trees: 1.58±0.13 cm *vs*. 1.72±0.15 cm (*t* = 0.62; *p* = 0.50), and number of leaves: 22.7±1.3 *vs*. 23.8±1.9 (*t* = 0.50; *p* = 0.61). Because *Hirtella* trees are patchily distributed (several individuals within a 3–5 m radius), we randomly allocated trees within each patch with respect to the treatment (giving us the final ratio of 41 control and 31 experimental trees). This keeps the majority of either experimental or control trees from being selected in the same zone since differences in the amount of ^15^N in the soil between zones can occur with repercussions for the δ^15^N of the plants.

Twenty-four trees (height: 1–1.8 m) that had lost their associated ant colonies before we began the experiment were taken into consideration for certain comparisons. Over the 9-month study, only some queens began to found colonies on 11 trees, but no workers patrolled the foliage. Other trees from this hill were not selected because they were either too small to produce flowers (<0.9 m), too tall (over 1.9 m) to be easily paired, or were in the process of regenerating after being broken by a branch that fell from the canopy.

Each month we counted the number of leaves, flower buds, flowers and fruits on both control and experimental trees. We were therefore able to deduce the number of new leaves produced by each tree as well as its level of flower and fruit production.

### Isotopic Analysis

Nitrogen exists in two stable (non-radioactive) forms, ^14^N and ^15^N, and the isotopic nitrogen composition of animal tissue reflects the isotopic ratio of food eaten with a ^15^N enrichment of 3–5‰ at each trophic level [29]. Also, the δ^15^N in plant tissue reflects the δ^15^N value of the nitrogen source. Therefore, if ants supply their host myrmecophyte with nutrients, we reasoned that plants whose associated ants were experimentally provided with surplus prey must be richer in ^15^N than those in the control treatment.

**Figure 2:**
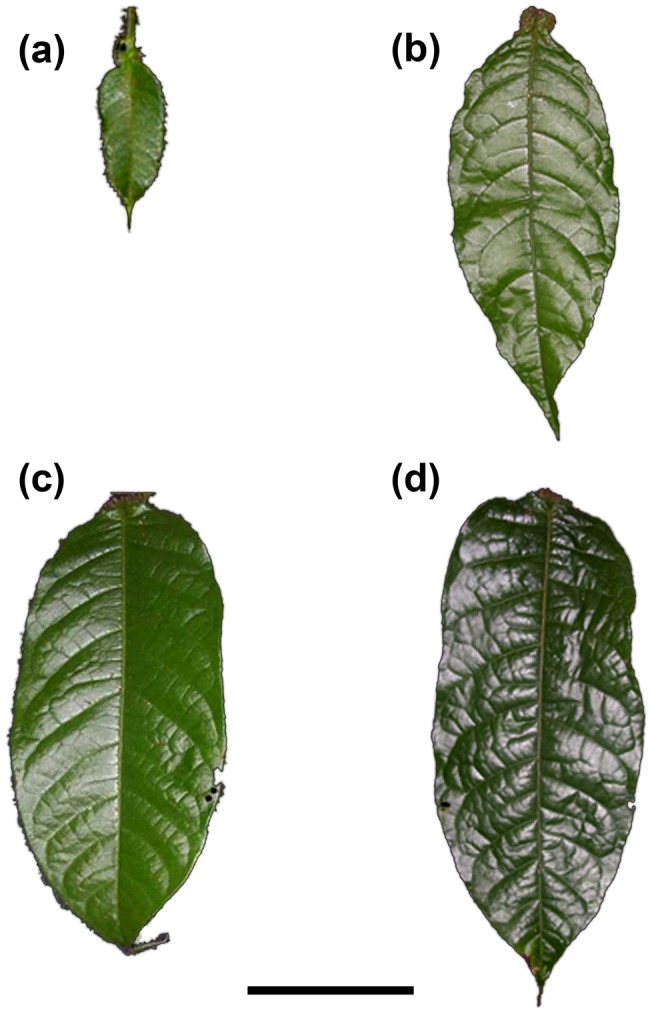
Four developmental stages of *Hirtella physphora* leaves. (a) Juvenile leaves: less than 5 cm long, positioned vertically; the domatia are not fully developed. (b) Expanding leaves: have fully developed domatia, but the blade is still immature. (c) Young leaves: from 15 to 25 cm long; positioned horizontally, mature but still relatively tender, and light green in color. (d) Old leaves: older, non-senescent, mature leaves of up to 30 cm in length, positioned horizontally, stiff, and dark green in color. Scale bars represent 5 cm.

At the end of the experiment, a 4-cm^2^ piece of a “young, well-developed leaf” (see “stage 3” leaf in [30] and in Fig. 2) was harvested from each host tree, freeze-dried and then ground into a homogeneous powder using a mixer mill. Stable isotope analyses on these plant samples were conducted at the Scottish Crop Research Institute, Invergowrie, Dundee, DD2 5DA, Scotland, UK, using a Thermo-Finnigan Delta^plus^ Advantage gas isotope-ratio mass spectrometer interfaced with a Costech Analytical ECS4010 elemental analyzer. The natural abundances of ^15^N were calculated as follows: 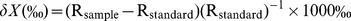
where *X* is the element of interest and R_sample_ and R_standard_ the molar ratios (i.e., ^15^N/^14^N) of the sample and the standard, respectively [29].

We conducted this study because we needed to be sure that the δ^15^N of the experimental trees had truly increased compared to the control trees.

### Biotic Protection Of Trees

Foliar growth is slow in *H. physophora*, and leaves can live for several years. While juvenile leaves benefit from the intense biotic protection provided by mutualistic ants, old leaves have their own efficacious mechanical and chemical defences [30]. So, we compared the defoliation rates of the two other kinds of leaves (“stage 2” and “stage 3”, respectively, according to Grangier *et al*. [30]; see Fig. 2) that were likely produced during the survey period: “expanding leaves” (“stage 2”) had fully developed domatia and an immature blade 8-to-15-cm-long (to be distinguished from “juvenile leaves” whose domatia were not fully developed), and “young, well-developed leaves” (“stage 3”).

At the end of the survey 28 control trees, 25 experimental trees (among the 41 and 31, respectively, from the beginning) and the 24 trees that had naturally lost their ants before the survey period bore such leaves, enabling us to compare the levels of defoliation using the following scale: (1) intact leaves or less than 5% of the leaf surface destroyed; (2) between 5% and 25% of the leaf surface destroyed; (3) between 25% and 50% of the leaf surface destroyed; (4) between 50% and 75% of the leaf surface destroyed; and (5) more than 75% of the leaf surface destroyed. We assigned the values 0, 1, 2, 3 and 4 for these levels of defoliation and we averaged these numbers *(∑defoliation values × number of leaves* ^−1^) to obtain a rate of herbivory per tree.

### Ant Castes Produced In Control And Experimental Colonies

To preserve some *H. physophora* plants in the area at the end of the survey, we gathered colonies from only 23 control and 23 experimental trees so that some intact trees and colonies remained to re-colonize the area. Indeed, to gather the ant colonies, we were obliged to cut off most of the host tree branches to collect both the leaf pouches (so the entire leaf) and the galleries where numerous workers were hiding and quickly placed them into plastic bags containing 0.5 L of 96% ethanol. We placed a label identifying the tree inside each bag, closed the bag, and then tagged the outside of the bag with the same code. The bags were then transported to the laboratory to quantify the size of the ant colony on each *H. physophora* tree.

All of the colonies had a queen. In the laboratory, we counted the number of males, male pupae, winged females, female pupae and larvae. For the production of workers, we could not consider the adult individuals as some of them likely emerged before the experimental period, so that we estimated the number of workers produced only from the number of pupae and larvae. We evaluated the number of worker larvae using the following formula: 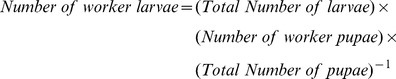 where *Total Number of pupae*  =  *male pupae + female pupae + worker pupae.*

### Statistical Comparisons

The Student's t-test was used each time a comparison of two sets of data was necessary. The levels of defoliation between treatments were compared using the Kruskal-Wallis test followed by a Dunn's *post-hoc* test. The link between the treatment and the numbers of leaves, buds or fruits on each plant was modelled using a generalized linear model (GLM) with a log-Poisson link [31]. The significance of the effect of the treatment was assessed through Likelihood ratio tests. Using the same statistics, the link between thetreatment and the number of buds that developed into flowers was modelled for each plant to obtain the transition rates from bud to flower; the same was done for the number of flowers that developed into fruits (R v. 2.14.2 software).

## Results

The numbers of workers and males produced by the experimental and control colonies during the survey period were not significantly different (means ± s.e.; workers: 167.60±13.24 *vs*. 174.40±19.36, *t* = 0.289, *df*  = 44, *p* = 0.77; males: 5.09±1.17 *vs*. 4.61±1.12, *t* = 0.294, *df*  = 44, *p* = 0.77). However, over-provisioned colonies produced significantly more winged females than control colonies (3.17±0.97 *vs*. 1.00±0.34, *t* = 2.24, *df*  = 44, *p* = 0.03).

The very similar rates of defoliation for experimental (median, 25% and 75% percentiles: 1.00, 0.50, 1.33) and control trees (1.00, 0.62, 1.00) were much lower than for trees having lost their guest ant colony (3.33; 3.00; 3.41, Kruskal-Wallis test: *H*_2,77_  = 47.22, *p*<0.0001; Dunn's *post-hoc* test: rates of defoliation for experimental *vs*. control trees, not significant; experimental and control trees *vs*. trees having lost their guest ants, *p*<0.001). Among the 24 trees that had naturally lost their ants before the survey, six produced inflorescences and a total of 26 flower buds. Defoliating insects destroyed 21 of these buds as well as the five flowers produced.

Both plant growth and reproductive investment were enhanced by over-provisioning the ant colonies as the experimental trees produced significantly more leaves (3.58±0.40 *vs*. 2.17±0.31, *p*<0.001), more flower buds (5.81±1.45 *vs*. 3.27±0.64, *p*<0.0001), more flowers (3.61±0.83 *vs*. 1.78±0.39, *p*<0.0001) and more fruits (1.90±0.50 *vs*. 0.49±0.19, *p*<0.0001) than control trees (Likelihood ratio test on the GLM-Poisson model; R statistics). The percentage at which flower buds matured into flowers was not significantly different between experimental and control trees (64.3±5.5 % *vs*. 49.9±7.3 %, *p* = 0.48 for the Likelihood ratio test on the GLM-Binomial model). So, it is likely that the workers castrated their host trees regardless of whether the trees belonged to the experimental or the control group. Finally, the percentage of flowers that matured into fruit was significantly higher for experimental than for control trees (49.7±5.4 % *vs*. 23.3±6.0 %, *p* = 0.02 for the Likelihood ratio test on the GLM-Binomial model).

**Figure 3:**
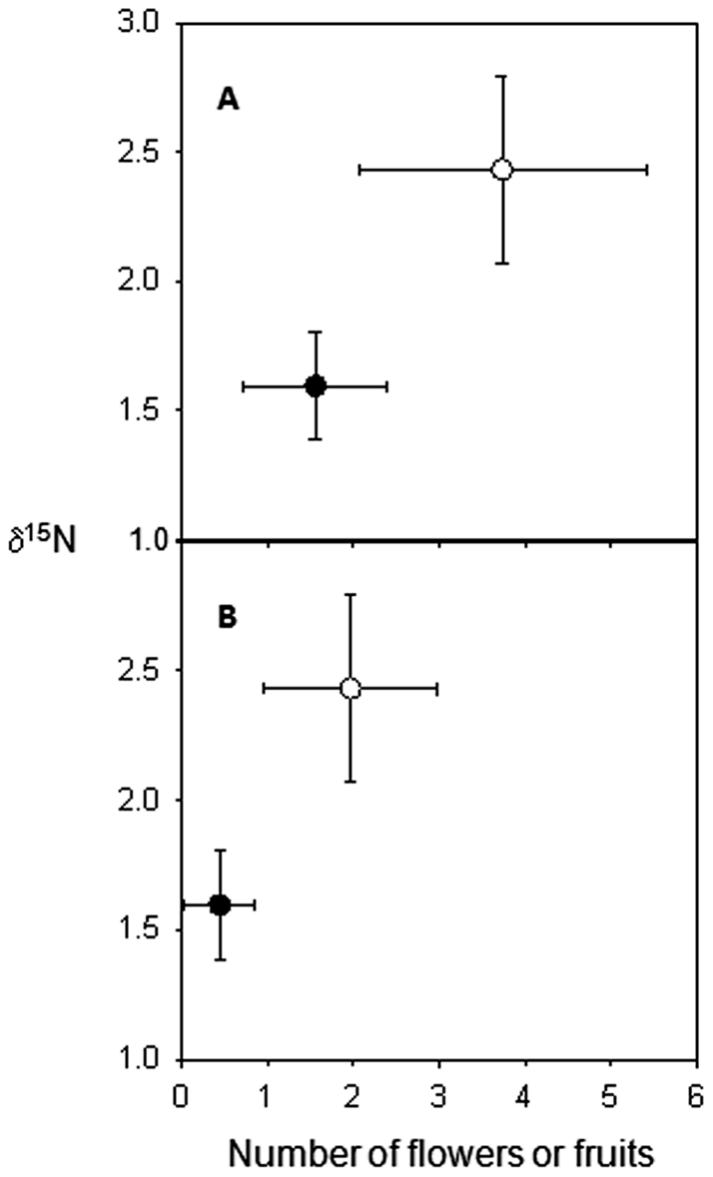
Production of flowers (A) and fruits (B) *versus* δ15N values (‰) for the leaves of *Hirtella physophora*. The resident *Allomerus decemarticulatus* colonies were over-provisioned (experimental trees; open circles; N = 31) or not (control trees; filled circles; N = 41) (means ± se).

At the end of the survey, while the percentage of nitrogen contained in the leaves of both experimental and control trees was not significantly different (mean ± SE: 1.46±0.03 % vs. 1.42±0.02 %; *t* = 0.95; *df*  = 70; *p* = 0.35), the experimental trees had significantly higher δ^15^N values than control trees (2.45±0.18 ‰ *vs*. 1.59±0.11 ‰; Welch-corrected *t* = 4.08, *df*  = 51, *p*<0.001; see Fig. 3).

## Discussion

Here we show that colony fitness was enhanced through over-provisioning with prey as more winged females were produced by experimental colonies. Supplemental protein increased the production of males and gynes, but not of workers, in the Argentine ant *Linepithema humile* [32], while the increased production of winged females was due to supplementary carbohydrates rather than proteins in *Myrmica brevispinosa* [33].

The experiment did not modify the worker patrolling activity because we noted non-significant differences in the: (1) number of workers produced per colony sheltered, (2) biotic protection (similar defoliation rate; having very low constitutive defences, the leaves at the chosen stages of development depend on ant protection [30]) and (3) castration rate between control and experimental trees.

On the plant side, experimental individuals had more ^15^N than did control plants, confirming findings by Leroy *et al*. [19] of myrmecotrophy in this mutualism. The novelty of this study is that “surplus” nutrients likely permitted the experimental trees to increase their investment in both growth and reproduction as they produced significantly more leaves, flower buds, flowers and fruits than control trees. Furthermore, the experimental trees had a higher rate of flowers that matured into fruits compared to the control trees, showing that fruiting is likely limited by the quantity of available nitrogen.

Our results support the initial hypothesis that the more insects the ants capture, the more nutrients are available to the host plant for growth. This provides the ants with more housing, in turn providing accommodation for the additional winged females produced and enabling the ants to build longer gallery-traps to capture more prey. In addition to this self-sustaining process, the prey carcasses incorporated into the trap to feed the fungus attract necrophagous insects that are then frequently captured [25], while the trapped prey attract cleptobionts that are also captured (Fig. 1a; [34]).

Cases of plant-ants sterilizing their host myrmecophyte have frequently been noted [20]–[24],[27]. This behaviour corresponds to the “over-exploitation” of the partner, increasing that partner's cost, as opposed to “cheating by defection”, or reducing the partner's benefit [35],[36]. In response, myrmecophytes can keep their guest ants from destroying flowers by changing the distribution of the inflorescences and through repellent floral volatiles [37],[38] or through retaliation where they destroy certain domatia or reduce their size and survival rate [39],[40]. For *H. physophora*, a trade off occurs between the partial castration carried out by the *A. decemarticulatus* workers [24],[27] and myrmecotrophy that indirectly favours both plant growth and fitness (this study). Although entirely dependent only on the ants' behaviour, these mechanisms seem to increase the stability of the relationship.

In conclusion, myrmecotrophy, a byproduct benefit for myrmecophytes, may intervene in the stability of myrmecophyte-ant mutualisms because the plant receives a benefit at no cost (or extremely reduced cost) for the ant. Further studies are needed to verify whether myrmecotrophy is more widely found than previously thought, particularly when the plant-ants are predatory and have a tendency to partially castrate their host plant.
